# Genetic evidence for the role of non-human primates as reservoir hosts for human schistosomiasis

**DOI:** 10.1371/journal.pntd.0008538

**Published:** 2020-09-08

**Authors:** Tadesse Kebede, Nicolas Bech, Jean-François Allienne, Rey Olivier, Berhanu Erko, Jerome Boissier

**Affiliations:** 1 Department of Microbiology, Immunology and Parasitology, School of Medicine, Addis Ababa University, Addis Ababa, Ethiopia; 2 Aklilu Lemma Institute of Pathobiology, Addis Ababa University, Addis Ababa, Ethiopia; 3 Laboratoire Interactions Hôtes-Pathogènes-Environnements (IHPE), UMR 5244 CNRS, University of Perpignan, IFREMER, Univ. Montpellier, F-66860 Perpignan, France; 4 Laboratory of Ecologie et Biologie des Interactions (EBI), UMR CNRS 7267, Poitiers University, Poitiers, France; Wellcome Sanger Institute, UNITED KINGDOM

## Abstract

**Background:**

Schistosomiasis is a chronic parasitic disease, that affects over 207 million people and causes over 200,000 deaths annually, mainly in sub-Saharan Africa. Although many health measures have been carried out to limit parasite transmission, significant numbers of non-human primates such as *Chlorocebus aethiops* (*Ch*. *aethiops*) (vervet) and *Papio anubis* (baboon) are infected with *S*. *mansoni*, notably in Ethiopia, where they are expected to have potentially significant implications for transmission and control efforts.

**Objective:**

The objective of this study was to assess and compare the genetic diversity and population structure of *S*. *mansoni* isolates from human and non-human primates free-ranging in close proximity to villages in selected endemic areas of Ethiopia.

**Methods:**

A cross-sectional study was conducted in three transmission sites: Bochesa, Kime and Fincha. A total of 2,356 *S*. *mansoni* miracidia were directly isolated from fecal specimens of 104 hosts (i.e. 60 human hosts and 44 non-human primates). We performed DNA extraction and PCR amplification using fourteen microsatellite loci.

**Results:**

At population scale we showed strong genetic structure between the three sample sites. At the definitive host scale, we observed that host factors can shape the genetic composition of parasite infra-populations. First, in male patients, we observed a positive link between parasite genetic diversity and the age of the patients. Second, we observed a difference in genetic diversity which was high in human males, medium in human females and low in non-human primates (NHPs). Finally, whatever the transmission site no genetic structure was observed between human and non-human primates, however, there appears to be little barriers, if any, host specificity of the *S*. *mansoni* populations with cross-host infections.

**Conclusion:**

Occurrence of infection of a single host with multiple *S*. *mansoni* strains and inter- and intra-host genetic variations was observed. Substantial genetic diversity and gene flow across the *S*. *mansoni* population occurred at each site and non-human primates likely play a role in local transmission and maintenance of infection. Therefore, public health and wildlife professionals should work together to improve disease control and elimination strategies.

## Author summary

Schistosomiasis is a chronic disease caused by flukes (trematodes). The definitive host spectrum of schistosomes, whether human, non-human primates (NHPs) or other mammals, is highly dependent on the schistosome species concerned. Genetic diversity and population structure studies of *S*. *mansoni* have provided insights into the variation of natural populations. Understanding *S*. *mansoni* genetic diversity and population structure of isolates from human and non-human primate hosts living in close proximity showed the occurrence of infection of a single host with multiple *S*. *mansoni* strains and inter- and intra-host genetic variations. In this article, the researchers assert the fact that genetic approach reveals that parasites from the three different sites are independent. Thus, we could consider the three sites as geographical replicates showing the influence of NHPs in parasitic transmission in Ethiopia. This study provides insights into the epidemiology, genetic diversity and population structure of *S*. *mansoni* in human and non-human primates in Ethiopia, all of which are crucial for the control of schistosomiasis.

## Introduction

Schistosomiasis remains one of the most prevalent and debilitating helminthic infections in the world. Globally more than 207 million people are infected, and over 600 million people are at risk of infection [1]. These parasites have a complex life cycle with freshwater snail intermediate hosts and vertebrate definitive hosts. Like other trematodes, schistosomes are characterized by a certain specificity for their snail intermediate hosts. However, the definitive host spectrum of the 26 Schistosoma species varies greatly according to the species concerned. The ancestral *S*. *japonicum* species is characterized by a broad host range and the host specificity seems to be a derived characteristic with some species such as *S*. *intercalatum* recorded to only infect humans [2]. *Schistosoma mansoni* is present in South America and in Africa and it is responsible for the mesenteric form of the disease. Compared to *S*. *japonicum*, *S*. *mansoni* is less well recognized as a zoonotic parasite. We have identified evidence for 51 species being naturally infected with *S*. *mansoni* (S1 Table). It is important to notice that the majority of the identifications we have recorded in the database are based on egg morphology and not molecular identification. However, lateral spine eggs are not frequent in *Schistosoma* species and mis-assignation is unlikely. A bad assignation could concern *S*. *rodhaini*, a rodent parasite that produce eggs similar in shape to those of *S*. *mansoni*. With 59% and 24% of the species inventoried, the majority of non-human *S*. *mansoni* definitive hosts are rodents and primates, respectively. With human population growth or human-made landscape changes it is likely that non-human hosts could maintain the infection in nature and serve as sources of infection for humans (*i*.*e*. could act as reservoir hosts) and may have potentially significant implications for transmission and control efforts. Large-scale control programs are mainly based on morbidity control through a mass-drug chemotherapy program using a single drug treatment [3]. Despite their important role in the transmission of the parasite, the reservoir hosts are currently excluded from control programs in Africa. In China, an integrative control strategy that includes a mass-chemotherapy program and which also considers bovine hosts has proven to be highly efficient in reducing the prevalence of *S*. *japonicum* [4].

With close geographical and genetic proximity of many species of non-human primates (NHP) to humans, and given sufficient epidemiological opportunity, NHP can become infected with *S*. *mansoni* and may potentially exhibit similar clinical signs and symptoms [5] and would act as fully competent definitive hosts [6, 7]. Experimental studies conducted in the 1960s and 1970s comprehensively demonstrated that several NHP were able to maintain transmission of schistosomiasis and maintain the parasitic cycle [8–10]. Several field surveys have demonstrated that NHP can naturally host *S*. *mansoni* [11]. Natural *S*. *mansoni* infections have been evidenced in grivet monkeys (*Chlorocebus aethiops*) in Ethiopia [12–15], Kenya [16, 17] and Tanzania [14], in patas monkeys (*Erythrocebus patas*) in Cameroon [18], in chimpanzees (*Pan troglodytes*) in Nigeria [19] and Senegal [20], and in baboons (*Papio sp*.) in Saudi Arabia [21, 22], Ethiopia [13, 15, 23], Tanzania [24–26], Kenya [17, 27–29] and Nigeria [30]. Although baboons in many locations have been shown to be infected with *S*. *mansoni*, other sympatric NHP species were described as free of the parasite. For example, in Kenya *S*. *mansoni* infection was identified in baboons but not in local vervet or sykes monkeys [27]. Similarly, Legesse and Erko [23] observed *S*. *mansoni* infection in baboons in Ethiopia, but not in sympatric vervet monkeys. In several of these NHP schistosome infections, it has been suggested that forest fragmentation, increased proximity of humans to wild habitats and the emerging reliance of wild primates on human settlements for food, are at least partially responsible for increased exposure and risk of these animals contracting ‘human’ diseases [30]. This is mainly due to the rapid population growth rate and human-mediated landscape changes. A worrying trend is that national park and forest reserve areas, which might have been expected to allow for a degree of protection against zoonotic transmission of infections, also seem to show signs of parasite transfer from humans to animals, as has been seen in Mahale Mountains National Park and Gombe Stream National Park [5].

A multi-host parasite system does not automatically imply a reservoir host for the human parasite. Parasite and/or host behavior may limit the encounter of animal and human infecting pathogens. For instance, the pattern of cercarial emergence, a genetically controlled behavior, can limit parasite exchanges between different host species. This has been demonstrated for *S*. *japonicum* where two distinct patterns have been identified according to the animal reservoir host implicated in the transmission: with parasites from rodent hosts emerging in the afternoon and those from cattle emerging in the early morning [31]. Interestingly, this phenotypic differentiation is based on clear genetic differentiation [31]. Population genetic tools, to accurately identify the species of the parasite are needed and, are powerful techniques to identify parasite gene flow in a multi-host parasite system [32]. This has been illustrated for *S*. *japonicum* where in villages of the Anhui province in China, different parasite lineages were identified between water buffalo or human hosts and goats, pigs, dogs or cats [33]. Similarly, close genetic proximity was observed between parasites recovered from dogs and humans in the Philippines [34]. Parasite reservoir hosts and subsequent control measures are thus dependent on the transmission site [35]. In Ethiopia, 30 million people are at risk of infection, while 4 million are already infected with *S*. *mansoni* or *S*. *haematobium* [36]. Although natural NHP infection with *S*. *mansoni* has been reported in Ethiopia, the precise role in the transmission has never been inferred. In this study, we investigated the genetic diversity and population structure of *S*. *mansoni* from humans and NHPs in three selected endemic localities. The study will provide insights into the definitive host range and transmission dynamics of *S*. *mansoni* between humans and NHPs, and will deliver important information for schistosomiasis control in Ethiopia.

## Materials and methods

### Study sites

**Fig 1:**
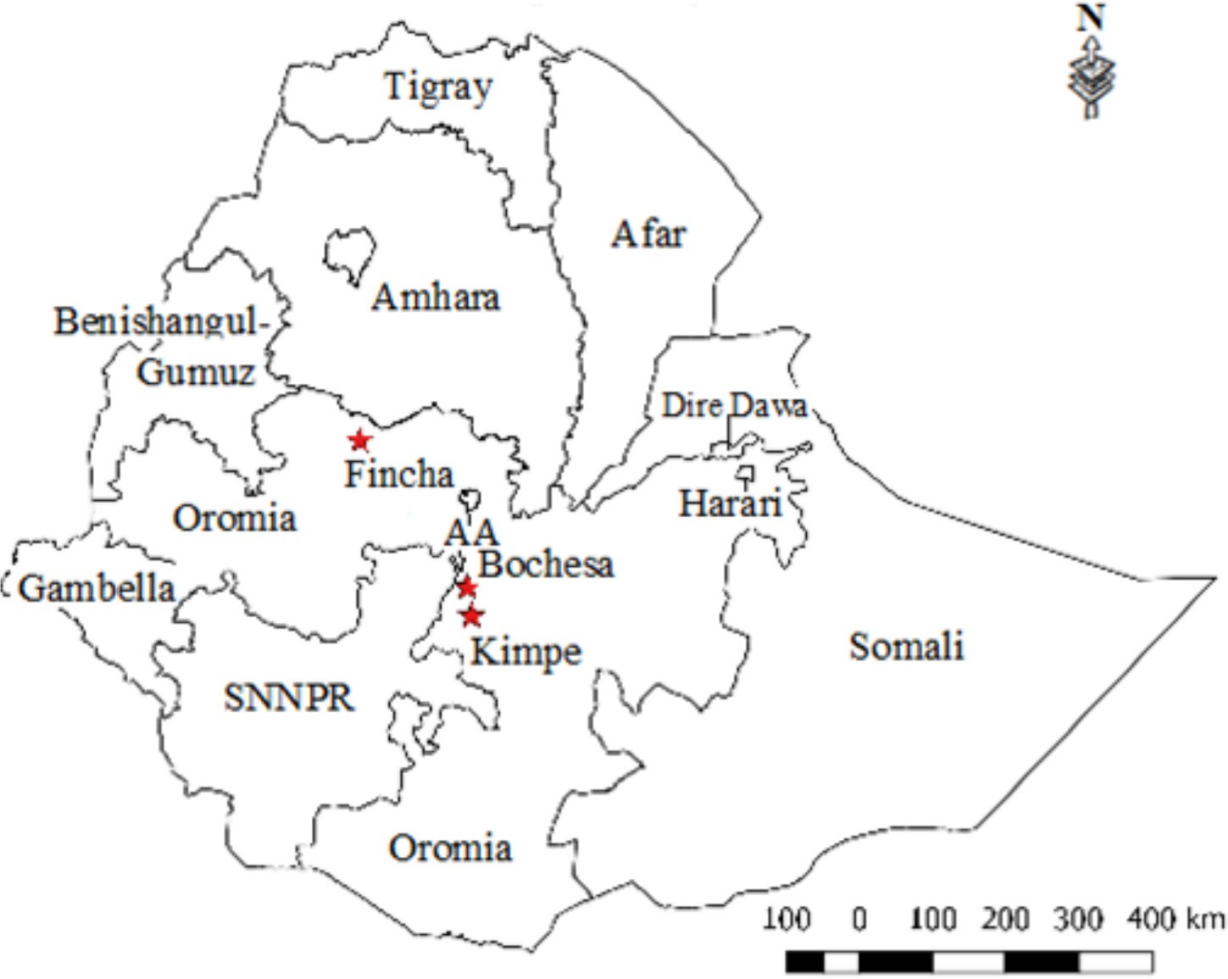
Map of Ethiopia showing study sites.

A cross sectional study was conducted in Bochesa village, Kime area, and Fincha (Fig 1) located in Oromia Regional State within previously reported endemic foci of *S*. *mansoni* [23, 37]. Bochesa village is located around Lake Ziway about 160 km south of Addis Ababa at an altitude of 1642 m above sea level. The people in the village use the lake water for irrigation, fishing and other domestic activities. Numerous vervet monkeys range in the environment and share the water source with humans. Kime is located 235km south of Addis Ababa on the eastern shore of Lake Langano. It is situated at about 1600m above sea level. There were two troops of free-ranging baboons with overlapping territories in Bishan Gari and Burka Dita forest reserve areas in close proximity to human habitation. The baboons range freely over human peridomestic habitats. The study area was previously described in detail [38]. Fincha Camp 7 site is located in the Fincha River Valley Sugar Plantation about 350km west of Addis Ababa at an altitude of about 1567m above sea level. The irrigation system of the area creates favorable conditions for transmission of schistosomiasis as a result of agro-industrial activities and high population influx [39]. There were also free ranging vervets and baboons living in the forest surrounding the sugar plantation. The prevalence of *S*. *mansoni* infection in human and NHP were previously published and synthetized in Table 1. This last study showed higher prevalence in male compare to female patients.

**Table 1:** *Schistosoma mansoni* prevalence in human and non-human primates in three transmission sites in Ethiopia (Kebede *et al*., 2018).

	Human		Vervet monkey	Baboon
	Male	Female		
Bochesa (n = 283)	30.3%* (n = 61)	7.3% (n = 6)	21.6% (n = 37)	
Kime (n = 318)	20.6%* (n = 28)	9.9% (n = 18)		51.7% (n = 58)
Fincha (n = 310)	33.1% (n = 58)	28.9% (n = 39)		45.5% (n = 11)
Total (n = 911)	28.7%* (n = 147)	18.8% (n = 63)		

The sample size is shown in the parentheses.

* Significant difference between male and female prevalence.

#### Stool sample collection

For humans, small plastic sheets were provided to study participants and a stool specimen was collected and examined microscopically using Kato-Katz method. *S*. *mansoni* positive egg specimens were kept in 0.85% saline solution and transported to the Medical Parasitology Laboratory of Aklilu Lemma Institute of Pathobiology, Addis Ababa University, to harvest miracidia. The age and the sex of each patient (*i*.*e*. host) were recorded [37].

For NHPs, fresh fecal droppings were collected early in the morning upon defecation and while the baboons were in the trees where they spent the night [23]. Microscopic examination was conducted and *S*. *mansoni* positive samples were kept in 0.85% saline solution and transported to the same laboratory for miracidia hatching. Samples were collected once from baboons from Kime and twice from Fincha over two separate days. Samples were collected twice from vervets in Bochesa, at two different locations and at different times. During collection, much care was taken to select only distinct droppings so that each dropping would represent a single baboon or vervet monkey sample. This sampling method limit our investigations because contrary to human samples we cannot know precisely which sample collected from which individual NHP.

#### Miracidium collection on FTA cards

For *S*. *mansoni* miracidium isolation, samples from each infected host were prepared separately. The stool specimens and fecal droppings were suspended in dechlorinated water and filtered through tiered sieves of different mesh sizes (425μm, 180μm and 140μm) and kept for about 20 min in order to allow the eggs to settle in the bottom of the flask. The eggs were washed twice by filling the flask with distilled water and then allowing them to rest submerged for 30 minutes. Parasite eggs concentrated at the bottom of the flask were kept in the dark for 30 minutes and hatching was induced by exposure to bright light on a Petri dish. Miracidia were individually pipetted both for human and NHPs onto the Whatman FTA Elute card using a dissection microscope [40].

#### DNA extraction

A 4.0mm disc was removed with micro-punch from the FTA Elute Card where the miracidia sample was loaded and put in a tube. 50μl Milli-Q water was added to each tube, incubated at room temperature for 10 minutes and then removed. 100μl of 5% (wt/vol) Chelex 100 solution was pipetted into each tube while the resin beads were evenly distributed (gentle mixing with a stir bar) in solution using a large bore pipette tip [41] followed by incubation at 56°C for 20 minutes and at 99°C for 8 minutes. The tubes were centrifuged at 12,000 rpm for 2 minutes and the supernatants (schistosome genomic DNA) were put in a 96 well plate until PCR processing.

#### PCR amplification and genotyping

Fourteen previously published polymorphic microsatellite markers were chosen based on their consistent and reproducible amplification under standard conditions and used in two multiplexes (details of loci are provided in Table 2). Multiplex one contains loci SMDA28, S9-1, SMD28, SMD25, SMD89 and SMDO11, and multiplex two contains loci CA11-1 and SmBr10, BF936409, SmC1, SmBr1, SmBr16, R95529 and SmBr5. PCR was performed in a total volume of 10μl containing 5μl of 2x Master Mix QIAGEN which contains: HotStarTaq DNA Polymerase, PCR Buffer (6 mM MgCl2, pH 8.7 (20°C), dNTP mix (dATP, dCTP, dGTP, dTTP), 1μl Primer mix and 4μl schistosome genomic DNA. The cycling reaction was done in a programmable thermal cycler (TC-Plus, TECHNE, UK) set to heat at 95°C for 15 min initial activation, followed by 40 cycles at 94°C for 30 s (denaturing), 56°C for 90 s (annealing), 72°C for 60 s (extension), and a final extension at 60°C for 30 min.

**Table 2:** Primer sequences and characteristics of *Schistosoma mansoni* microsatellite loci used in this study.

Locus	Accession No.	Multiplex	Primer sequences (5′–3′)	Dye	Size bp	Repeat	Ref.
smda28	AF325695	1	F = CATGATCTTAGCTCAGAGAGCC R = AGCCAGTATAGCGTTGATCATC	VEC	91–115	(GATA)7-14	[43]
S9-1	AF330106	1	F = ATTACGATTGCACAGATACTTTTG	VEC	198	(GT)16	[44]
			R = TTTCAGAAATTTGTT TCCTCCTC				
SMD28	AF202966	1	F = CATCACCATCAATCACTC	VEC	240–244	(CAA)_5_	[45]
			R = TATTCACAGTAGTAGGCG				
SMD25	AF202965	1	F = GATTCCCAAGATTAATGCC	NED	292	(CA)_10_	[45]
			R = GCCATTAGATAATGTACGTG				
SMD89	AF202968	1	F = AGACTACTTTCATAGCCC	PET	153	(TC)_8_	[45]
			R = TTAAACCGAAGCGAGAAG				
SMDO11	AF325698	1	F = TGTTTAAGTCGTCGGTGCTG	PET	303–367	(GATA)_20−37_	[43]
			R = ACCCTGCCAGTTTAGCGTAG				
CA11-1	AI740374	1	F = TTCAAAACCATGAGCAATAGATAC	FAM	198	(AC)23	[44]
			R = CAACAAACAAGAAGGCTGATTAG				
SmBr10	DQ448293	2	F = CATGATCTTAGCTCAGAGAGC	FAM	109–133	(GATA)_10_	[46]
			R = GTACATTTTATGTCAGTTAGCC				
BF936409	BF936409	2	F = CACCTCAACACCTATG	FAM	224	(AAT)_13_	[47]
			R = GTTGGAAACACATTGGGC				
SmC1	AF325694	2	F = TGACGAGGTTGACCATAATTCTAC	FAM	287–296	(ATT)_6-16_	[43]
			R = AACACAGATAAGAGCGTCATGG				
SmBr1	L81235	2	F = GAGTATACGGCTTCTTGGA	PET	154	(AC)_9_	[48]
			R = CGGAACGACAAGAAAATCAT				
SmBr16	L04480	2	F = TGTGACTTTGATGCCACTGA	PET	337–341	(TA)_10_	[46]
			R = GGCCTGATACAATTCTCCGA				
R95529	R95529	2	F = GTGATTGGGGTGATAAAG	NED	228–275	(CAT)_10_	[45]
			R = CATGTTTCTTCAGTGTCC				
SmBr5	L25065	2	F = GAATTACTGTCCCTTTATCTC	NED	328	(ATT)7	[48]
			R = AAACTATTCATTACTGTCGGG				

Multiplexed PCR products were genotyped by a subcontractor (Genoscreen) and scored with GeneMapper v. 2.2 software. All genotype calls were manually identified and rechecked by a second reader for consistency. Of the 2,476 genotyped individuals, 2,354 contained adequate information which could be included in further analyses. Here, we tested the divergence from Hardy–Weinberg expectations using procedure implemented in the FSTAT v.2.9.3.2 [42] and based on 10 000 randomizations. In the same way, linkage disequilibria were tested using the procedure implemented in the FSTAT v.2.9.3.2 [42]. This option allowed testing the significance of association between genotypes by estimating p-values from randomizations of genotypes [42], p-values threshold were adjusted using Bonferroni correction.

#### Genetic diversity

Miracidia from all hosts (human or NHP) within a single site (Bochesa, Kime or Fincha) were treated as a population while all miracidia from a single host were treated as an infrapopulation. The genetic diversity of the six schistosome populations and all infrapopulations were assessed by measuring the expected heterozygosity (He), the allelic richness (Ar) and the inbreeding coefficient (*Fis*) at each microsatellite locus and for each population using Fstat software 2.9.3.2. The most likely numbers of breeding males and females within an individual host were assessed using Colony software v. 2.0.5.8 assuming random-mating population [49]. Only infrapopulations with a minimum of 10 *S*. *mansoni* larvae were included in this sibship analysis. In each site, expected heterozygosity (He) and allelic richness (Ar) were compared between the parasite populations recovered in humans and NHP using pairwise t-tests using SPSS 20.0. The age of hosts, the number of breeders, the expected heterozygosity (He) and the allelic richness (Ar) were compared using ANOVA followed by LSD pos-hoct tests. Pearson correlation tests were performed to assess a possible link between the age of the human host and the infrapopulation genetic diversity indexes (He, Ar, *Fis* and number of breeders). Finally, in order to infer possible relationship between variables (age and sex) we built full factorial general linear models for each genetic diversity indexes with sex as an explanatory categorical variable and age as a continuous co-factor.

#### Population genetic structure

Genetic differentiation was first measured by estimating the *F*_*ST*_ values between all pairs of populations [50] (The p-value estimated after 300 permutations and indicative adjusted nominal level (5%) for multiple comparisons was 0.003333). Treating miracidia as independent individuals, we investigated genetic structure using both Factorial Correspondence Analysis (FCA) implemented in Genetix software and Bayesian approach using Structure software 2.0 [51]. The FCA analysis allowed us to visualize the distribution of the genetic variations within and among populations. The Bayesian approach allowed for the assignment of individuals to a user-defined number of clusters (K). The maximum probability value of K was obtained by analyzing the likelihood of the data for different values of K from 1 to 7 using the Delta K method implemented in Structure harvester software [52]. Each simulation was replicated 20 times [53]. In this analysis, we chose the admixture model and the option of correlated allele frequencies among populations. The number of iterations was 1,000,000 and the burn-in period was 10,000.

### Ethical clearance

Ethical clearance was obtained from the Institutional Review Board (IRB) of Aklilu Lemma Institute of Pathobiology, Addis Ababa University (Ref. No.: ALIPB/994/2005/13) and the National Research Ethics Review Committee of Federal Republic of Ethiopia Ministry of Science and Technology (Ref. No.: 3.10/434/106). All ethical issues were strictly handled as per the International Ethical Guidelines for Biomedical Research. Informed verbal consent was obtained from all adults because the study participants provided only stool specimens and the procedure was not invasive presenting no more than minimal risk of harm to the study participants. Hence, the IRB approved the use of oral consent as we requested. For children younger than 18, informed verbal consent was obtained from their parents and the children also gave their assent. During specimen collection, oral consent for all those who agreed was documented as consented. All study subjects found positive for *S*. *mansoni* and other intestinal helminth were treated with praziquantel at a dose of 40mg/Kg body weight and albendazole (400 mg), respectively. Non-human primates’ droppings sampling was initiated after explicit consent was obtained from local authorities.

## Results

### Database and genetic diversity

One hundred and four different parasite infrapopulations (60 from human and 44 from NHP) with a total number of 2,354 *S*. *mansoni* miracidia (Table 3), with an average of 22.7 (SD+ 8.4) larvae per host were successfully genotyped and analyzed using 14 microsatellite loci (Database in S2 Table).

**Table 3:** Number of human or non-human primate hosts (vervet monkey or baboon) sampled by transmission site (Bochesa, Kime and Fincha). Numbers in parentheses represent the total number of *Schistosoma mansoni* miracidia recovered.

	Human	Vervet monkey	Baboon	Total
Bochesa	22(619)	13(254)	-	35(873)
Kime	18(424)	-	26(599)	44(1,023)
Fincha	20(381)	-	5(77)	25(458)
Total	60(1,424)	13(254)	31(676)	104(2,354)

We found no evidence for linkage disequilibrium as no significant association between genotypes was detected (p-value threshold after Bonferroni correction, p-0.0005). At the population scale, 42 of 84 *Fis* values showed significant departures from Hardy Weinberg expectations (p-value threshold after Bonferroni correction, p-0.0036) with *Fis* values (ranging from -0.09 to 0.45; mean *Fis* values = 0.055), revealing an excess of homozygosity (Table 4). These results likely stem from a Wahlund effect as each population analyzed included miracidia from different hosts (*i*.*e*. infrapopulations). We detected no deviation from Hardy Weinberg equilibrium at the infrapopulation scale (ranging from -0.54 to 1; mean *Fis* values = 0.02) (S3 Table). At the population scale, allelic richness ranged from 1.48 to 24.44 with a mean value of 8.57 (Table 4). This allelic richness was higher in the parasite population recovered from humans compared to the parasite population recovered in NHP for Bochesa (t = 2.80, p<0.05) and Fincha (t = 2.99, p<0.05) but not for the Kime population (t = -0.77, p>0.05). Allelic richness was lower in Kime compared to Bochesa and Fincha for both human and NHP (p<0.05). Expected heterozygosity (He) for all populations was 50.1%. No statistical difference was observed in He between parasites coming from human and NHP (pairwise t-tests p>0.05). Expected heterozygosity was lower in Kime compared to Bochesa and Fincha for both humans and NHP (pairwise t-tests p<0.05).

**Table 4:** Genetic diversity parameters for each locus and per population.

		Bochesa		Kime		Fincha		All
		Human	Vervet monkey	Human	Baboon	Human	Baboon	
SMDA28	Ar	10.89	9.20	4.89	4.61	9.04	9.70	11.42
	He	66.8%	72.1%	23.6%	21.4%	63.3%	69.7%	52.8%
	*Fis*	0.004*	0.004	0.081*	0.009	0.020	-0.039*	0.010
S9-1	Ar	5.03	6.06	3.74	3.72	5.35	5.00	6.58
	He	55.4%	54.0%	44.5%	46.0%	67.6%	66.8%	55.7%
	*Fis*	0.095*	0.140*	0.009*	0.012*	-0.002	0.189	0.075
SMD28	Ar	3.40	3.00	2.55	2.11	2.17	3.00	3.15
	He	31.3%	24.3%	45.5%	45.3%	7.7%	11.5%	27.6%
	*Fis*	0.056*	-0.008	-0.064*	-0.087	0.030	0.074	0.001
SMD25	Ar	6.37	5.39	5.60	4.93	9.60	6.89	7.13
	He	71.3%	68.1%	69.0%	67.7%	78.5%	79.0%	72.3%
	*Fis*	0.023*	-0.005*	-0.009*	0.001	0.016	0.076	0.014
SMD89	Ar	1.96	2.00	1.82	1.44	2.44	2.00	2.07
	He	4.4%	6.5%	2.3%	0.8%	19.8%	8.7%	7.1%
	*Fis*	-0.022	-0.033	0.192*	-0.003	-0.092	-0.041	0.000
SMD011	Ar	20.43	16.54	18.50	19.31	23.73	22.86	28.60
	He	92.4%	90.5%	87.5%	86.6%	91.7%	90.1%	89.8%
	*Fis*	0.075*	0.023*	0.057*	0.070	0.074*	0.036*	0.054
CA11-1	Ar	5.41	5.00	4.75	4.60	8.17	7.91	7.65
	He	64.2%	68.2%	58.2%	56.0%	67.5%	70.4%	64.1%
	*Fis*	0.094*	0.160*	0.082*	0.092*	-0.015*	0.053	0.077
SmBr10	Ar	10.62	9.38	3.79	3.91	8.61	7.89	11.47
	He	64.7%	72.1%	21.3%	18.3%	61.8%	69.7%	51.3%
	*Fis*	0.044*	0.071	-0.052*	-0.090*	0.033	0.012	-0.001
BF9364	Ar	4.31	3.90	2.82	2.45	7.15	4.00	5.06
	He	55.6%	55.1%	47.2%	44.2%	61.4%	55.7%	53.2%
	*Fis*	0.044*	0.010*	-0.019*	0.023	0.004*	-0.004	0.021
SmC1	Ar	4.00	3.00	3.44	3.22	5.42	4.00	4.48
	He	50.0%	50.7%	6.1%	7.6%	66.5%	57.8%	39.8%
	*Fis*	0.054	-0.026*	0.451*	0.061	0.032	0.075	0.106
SmBr1	Ar	3.72	3.25	1.83	2.18	3.53	3.00	3.86
	He	12.6%	14.7%	1.5%	2.0%	16.3%	31.8%	13.2%
	*Fis*	0.085*	-0.007*	0.331*	0.162	-0.011	0.113	0.109
SmBr16	Ar	13.10	11.27	9.50	11.16	7.76	7.74	13.46
	He	82.9%	83.6%	74.1%	77.1%	33.7%	24.4%	62.6%
	*Fis*	0.052	-0.033*	0.083*	0.094	0.074	0.089*	0.055
R95529	Ar	3.65	3.92	2.78	2.56	9.81	8.00	7.88
	He	28.0%	34.2%	48.3%	47.4%	81.7%	79.1%	53.1%
	*Fis*	-0.001*	0.120	0.007*	0.016*	-0.004	0.084	0.039
SmBr5	Ar	4.58	3.54	2.89	3.22	5.61	5.00	4.53
	He	67.4%	65.6%	51.5%	51.1%	57.1%	54.3%	57.8%
	*Fis*	0.192*	0.085*	0.229*	0.245	0.242*	0.358	0.215
All	Ar	6.96	6.10	4.92	4.96	7.74	6.93	8.38
	He	53.4%	54.3%	41.5%	40.8%	55.3%	54.9%	50.0%
	*Fis*	0.064	0.041	0.049	0.050	0.035	0.078	0.055

Ar: allelic richness based on minimum sample size of 66 diploid individuals, He: expected heterozygosity and *Fis* computed for each population using FSTAT ver. 2.9.3.2 software (1200 permutations) (Goudet 2001).

*:*Fis* with significant departures from Hardy–Weinberg expectations (*i*.*e*. significantly different from 0; P<0.0005 after Bonferroni adjustment).

**Fig 2:**
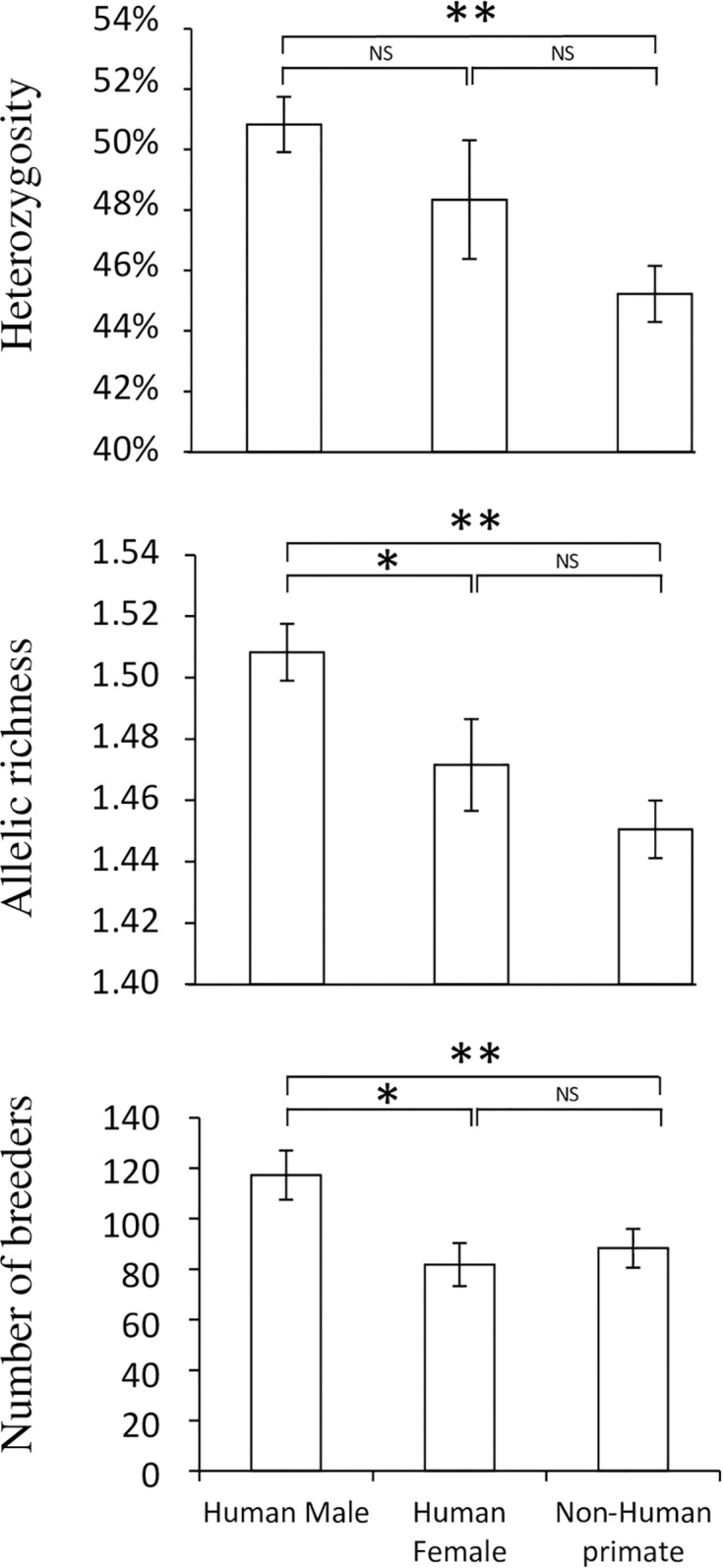
Mean (±Standard Deviation) expected heterozygosity, allelic richness and number of breeders in schistosome infrapopulations recovered from human males, human females and non-human primates in Ethiopia. Statistical difference was assessed using one-way ANOVA followed by LSD post-hoc test. *: statistical difference at 5% level. NS: not significant.

Genetic diversity indices (Ar, He, *Fis* and number of breeders) for each infrapopulation are presented in S3 Table and mean (±SD) values in Table 5. Mean values irrespective of the transmission site are presented in Fig 2. There is no difference in age between the group of female and male (Table 5). No statistical difference was observed in either the average age (t = 0.18, p>0.05) or in the distribution of age (Kolgomorov test Z = 0.74, p>0.05) according to the sex of the patient. The Fig 2 shows the genetic diversity indexes according to the type of host. We can notice a general trend, with the highest genetic diversity index in the parasite infrapopulations coming from human males, followed by human females and lastly, by NHP. Significant differences in He, Ar and the number of breeders were observed according to the type of host (F_2,101_ = 6.18, F_2,101_ = 7.87 and F_2,87_ = 4.36 for He, Ar and number of breeders respectively, p<0.05). Finally, we observed that the age of male patients was positively correlated to He (n = 36, r = 0.333, p<0.05), Ar (n = 36, r = 0.331, p<0.05), and the number of breeders (n = 32, r = 0.403, p<0.05) but not to the *Fis* (n = 36, r = -0.885, p>0.05) of the parasite infrapopulation they hosted. For female patients, no significant correlations were observed between genetic indices of the parasite infrapopulations they hosted whatever He (n = 24, r = 0.841, p>0.05), Ar (n = 24, r = -0.231, p>0.05), number of breeders (n = 20, r = -0.166, p>0.05) or Fis (n = 23, r = 0.013, p>0.05), and their ages (p>0.05). Full factorial generalized linear models (S4 Table) show no direct influence of the sex or the age of the patient but that the interaction between age and sex influence both Ar and the number of breeders but not He and the Fis. This result make sense considering that for these two parameters the correlation with the age is negative for female and positive for male.

**Table 5:** Mean (±SD) genetic diversity indices (He, Ar, Fis and number of breeders) for each parasite infrapopulation.

		Age			He			Ar			Fis			Number of breeders		
		N	Mean	SD	N	Mean	SD	N	Mean	SD	N	Mean	SD	N	Mean	SD
Bochesa	Human Male	18	19.78	1.75	18	0.524	.006	18	1.524	.006	18	0.095	.008	17	130.12	14.46
	Human Female	4	20.00	5.90	4	0.510	.012	4	1.510	.012	4	0.056	.029	4	92.25	29.04
	Non-Human Primate				13	0.506	.013	13	1.502	.015	13	-0.007	.012	10	40.90	9.10
Kime	Human Male	7	9.29	1.60	7	0.404	.005	7	1.404	.005	7	0.115	.030	7	73.14	10.72
	Human Female	11	15.45	3.02	11	0.402	.009	11	1.402	.009	11	0.071	.021	10	70.90	10.81
	Non-Human Primate				26	0.409	.003	26	1.408	.003	26	0.114	.010	25	105.32	8.49
Fincha	Human Male	11	11.45	1.83	11	0.540	.005	11	1.544	.005	11	-0.001	.021	8	128.38	16.27
	Human Female	9	11.67	2.59	9	0.572	.028	9	1.540	.013	9	0.400	.012	6	93.00	12.60
	Non-Human Primate				5	0.538	.019	5	1.536	.019	5	0.060	.012	3	104.33	19.53
All populations	Human Male	36	15.19	1.32	36	0.507	.009	36	1.506	.009	36	0.070	.012	32	117.22	9.73
	Human Female	24	14.79	1.95	24	0.483	.020	24	1.472	.015	24	0.057	.012	20	81.80	8.55
	Non-Human Primate				44	0.452	.009	44	1.450	.009	44	0.072	.012	38	88.29	7.70

### Population genetic structure

**Fig 3:**
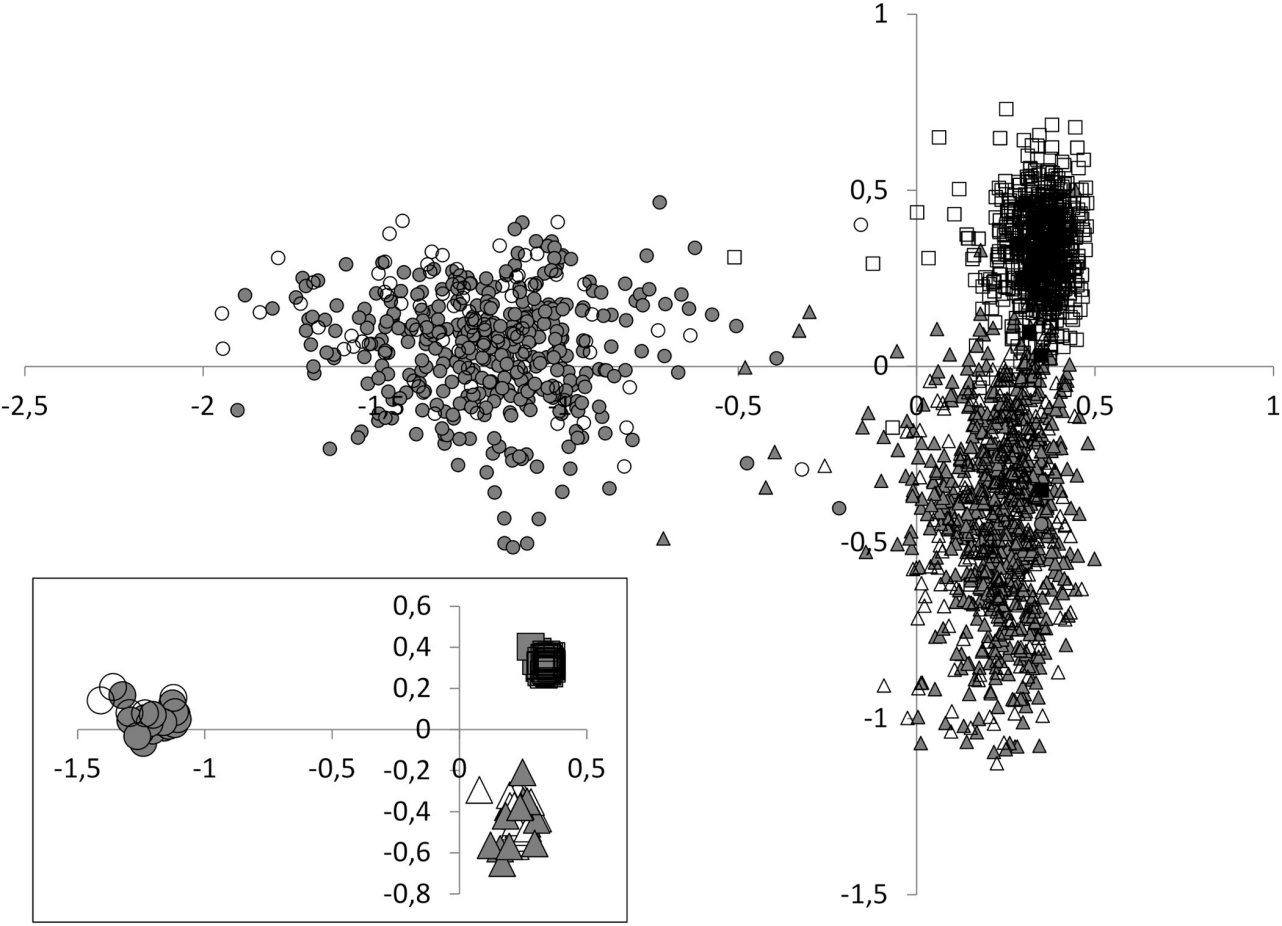
Results of Factorial Correspondence Analysis (FCA) showing the relationship among the six parasite populations. Each dot represents one miracidia. Circle for Fincha site, square for Kime and triangle for Bochesaa. Empty symbols represent miracidia originating from non-human primates and plain symbols represent miracidia coming from humans. The first and the second axes represent 34.44% and 11.80% of the genetic variation, respectively. In the box, each dot represents one host, with a plain symbol for humans and an empty symbol for non-human primates.

**Fig 4:**
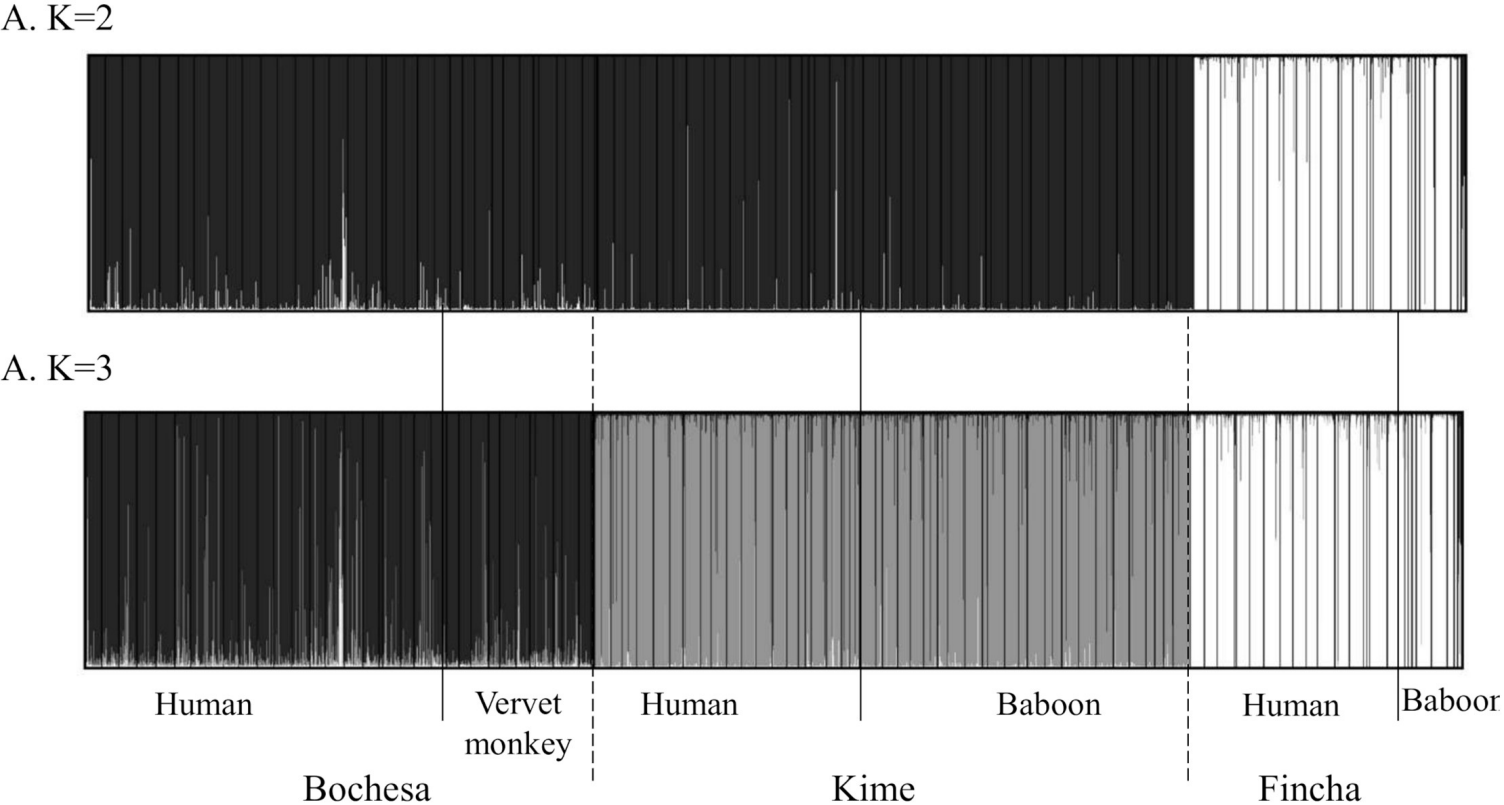
Population genetic structure of the 2,354 miracidia analysed with Structure software 2.0. The miracidia are represented into either K = 2 (A) or K = 3 (B) clusters. Each individual miracidium is represented by a thin vertical line, which is partitioned into a number of segments that represent the individual’s estimated membership fractions in the relative genetic clusters K.

All pairwise *F*_*ST*_ values (Table 6) were significant after Bonferroni correction (p-value threshold = 0.003). These *Fst* values follow an isolation by distance pattern with Fincha distant from the other two sites. The lowest *Fst* values were between human and NHP parasites irrespective of location. Pairwise *F*_*ST*_ values of *S*. *mansoni* between human and vervet monkey infections in Bochesa was 0.0066, between human and baboon infections in Kime was 0.0019, and between human and baboon infections in Fincha was 0.0083. On average, pairwise *Fst* values between parasite populations recovered in human and NHP was 0.056±0.003 compared to 0.160±0.080 and 0.175±0.092 between parasite populations recovered in humans and NHP in different foci, respectively. Results from FCA (Fig 3) showed a spatial genetic structure of *S*. *mansoni* miracidia characterized by three groups of points corresponding to the three geographical studied sites (*i*.*e*. Bochesa, Kime, and Fincha). While this main genetic structure distinguished miracidia infrapopulations from the different transmission sites, results within study sites, did not separate humans and NHP infrapopulations, which were totally admixed. This genetic structure was corroborated by an individual-based approach implemented in Structure software which inferred a highest posterior probability for two genetic clusters (i.e. K = 2) (Fig 4A and 4B).

**Table 6:** Pairwise genetic differentiation (*F*_*ST*_) among populations based on 14 microsatellite loci.

	Sample Source	Bochesa		Kime		Fincha	
Location		Human	Monkey	Human	Baboon	Human	Baboon
Bochesa	Human	-	*	*	*	*	*
	Monkey	0.0066	-	*	*	*	*
Kime	Human	0.0753	0.0816	-	*	*	*
	Baboon	0.0775	0.0845	0.0019	-	*	*
Fincha	Human	0.1713	0.1647	0.2333	0.2466	-	*
	Baboon	0.1798	0.1719	0.2534	0.2662	0.0091	-

*F_*ST*_ below diagonal and their significance level above diagonal (p<0.05)

However, the shoulder in the delta K curve at K = 3 (See S1 Fig) suggests the presence of a sub-structure organized into K = 3 genetic clusters (Fig 4A and 4B). This figure supports the FCA result that the Fincha population is more genetically distinct to the schistosome populations at the other two sites, but suggests a total lack of genetic structure between human and NHP parasites at each transmission site.

## Discussion

This study was conducted at three study sites (30 to 500 km apart) and shows a very strong spatial genetic structure of *Schistosoma mansoni* parasite populations originating from humans and non-human primates (NHP). According to the geographical distances between study sites, this significant genetic structure was expected. Similar results were previously found in Ethiopia [54], Kenya [55], Brazil [56], Uganda [57] and Senegal [58]. This pattern contrasts with that found for *S*. *haematobium* [59, 60] and *S*. *bovis* [61], where parasite populations show a weak genetic structure. Several external factors including intermediate host repartition, definitive host dispersal, host spectrum, drug pressure or internal factors such as effective population size or intrinsic genetic diversity could explain differences in the genetic structuring of the parasite population. We did not try to identify the eventual presence of *S*. *rodhaini* in the parasite we collected. Because, the microsatellite markers we used have been specifically designed on *S*. *mansoni*, even if cross species amplification is possible, we could expect to have some inefficient markers and/or a difference in allele size on *S*. *rodhaini* parasites. We can also expect to observe a population differentiation of these parasites. These last points have not been observed in the present study. However, considering the growing importance of hybrid schistosomes, including *S*. *mansoni x S*. *rodhaini* hybrids, we preconize to consider *S*. *rodhaini* in future studies.

We also observed that host factors are able to shape the genetic composition of parasite populations. First, we observed a positive relationship between parasite genetic diversity and the age of the host in male patients, with older men being infected with a more diverse parasite population. Second, we observed differences in genetic diversity between host categories, with high diversity in human males, medium in human females and low in NHPs. Host behavior and/or physiology is expected to shape the genetic composition of the parasite infrapopulation that they host. It has been proposed that the high genetic diversity of parasites is the result of host behavior in sampling several parasites in space and/or in time, resulting in ‘genetic mixing bowl’ for parasite genes [62–64]. A positive link between age of the patient and *S*. *mansoni* parasite diversity has already been observed in Senegal [64]. These authors showed that children were infected by more highly related parasites than adults. We can assume that this pattern is observable both in the present study and in the study of Van den Broeck *et al*. 2014, because both studies sampled a broad range of ages, including children and adults, while most other studies focused exclusively on children. A noticeable difference between our study and that of Van den Broeck *et al*. (2014) is that we did not observe this link for female patients, and Van den Broeck *et al*. did not observe differences between genders. Indeed, the sex of the host can also shape the genetic diversity of the parasite. Even if no link between the sex of the host and the genetic diversity of the harbored parasite was highlighted in Senegal [64–66], a subsequent study conducted in Ethiopia, revealed that males have a higher rate of infection and are more intensely infected than females [37]. For instance, higher *S*. *mansoni* genetic diversity has been observed in *Rattus rattus* males compared to females in the West indies [67]. The authors proposed different host behaviors (*i*.*e*. a difference in duration of cercariae recruitment or sex-biased dispersal and home range) or a difference in immunocompetence between sexes. The immunocompetence hypothesis has been experimentally tested and it has been shown that male mice are less susceptible to schistosome infection compared to females [68] and this could be due to a toxic effect of the testosterone on the schistosome larval stages [69]. These last experimental studies thus support the behavioral hypothesis over the immunocompetence hypothesis. Higher levels of *S*. *haematobium* genetic diversity has been observed in boys compared to girls in Zimbabwe [70]. In this last study, authors showed that males were more heavily infected than females because males had more contact with water [70].

Finally, the fact that NHPs harbor less parasite genetic diversity than humans could also result from behavioral differences including the frequency of water contact, which is much lower in NHPs than in humans. A general pattern can be proposed, establishing the link between host behavior (*i*.*e*. frequency of water contact), host immune defense mechanisms and parasite genetic diversity. The protective immunity hypothesis (also called ‘concomitant immunity’) states that the immune response stimulated by the adult parasite is more effective against new-coming larval schistosomes [71–73]. Experimental infection that used controlled infecting genotypes evidenced that the infectivity rate of incoming schistosomes is higher when the first infecting parasite is genetically dissimilar [74]. This genotype-dependent concomitant immunity will result in increasing genetic diversity of parasite infra-populations as the host is exposed to new-coming parasites. In the present study, differences in water contact frequency can be found according to the patient’s age, sex and species; these differences will in turn result in differences in parasite genetic diversity: high in human males, medium in human females and low in Non-Human Primates.

Even if our results reveal a primary genetic structure that is organized into three clusters which correspond to location (i.e. the 3 study sites), analyses (*i*.*e*. *Fst*) conducted between infrapopulations recovered in humans and NHP, revealed statistically significant genetic differentiations. However, these *Fst* values are less than 0.01 within the same study site and indicate a very low genetic differentiation between parasites harbored by humans and NHPs. This is consistent with NHP serving as reservoir hosts for human *S*. *mansoni* infections. Multi-host parasite systems have been well studied for *Schistosoma japonicum* because this parasite is recognized as a zoonotic parasite. In the marshland region in China, humans and bovines (cattle and water buffaloes) share the same *S*. *japonicum* genotypes but not with other definitive hosts (cats, dogs, pigs and goats) [33]. However, according to the transmission sites, the reservoir can change. For the same parasite species, in the Philippines, the reservoir host is the dog [34] and in the Hilly region in China the reservoir hosts are rodents and dogs [35]. Regardless of the reservoir host identified, the fact that the reservoir can change must be considered when planning control measures. Because current efforts are primarily based on mass chemotherapy programs, this approach may be ineffective if humans are re-infected by animal reservoir hosts. Our study provides genetic evidence and confirms that NHP play an important role in disease epidemiology of *S*. *mansoni* in Ethiopia. This is particularly relevant if we consider the high prevalence (21.6% to 51.7%) of NHP infections. Using barcodes, previous authors have also proposed that chimpanzees could act as reservoir host for *S*. *mansoni* [7, 75]. In many rural and recreational areas of Ethiopia, there is a close interaction between wild primates, especially baboons and vervets, and humans. In other areas with similar ecological conditions where schistosomiasis is endemic and in particular, where the number of water-based development projects are dramatically increasing, there is a need to assess the occurrence of natural schistosome infections in wild animals to avoid the risk of establishing zoonotic schistosomiasis. Whatever the reason, the involvement of freely ranging wild animals, such as monkeys and baboons, in the transmission cycle of schistosomiasis has a very important epidemiological implication in that it complicates control programs and it incurs additional control costs since it is difficult to treat wild animals even if the control program can financially afford treatment. However, controlling the Schistosome infection status of NHP reservoir hosts in not a trivial objective. First, it is not ethically conceivable to eliminate this kind of host and it is not easy to displace NHP populations. Second, even if several efficient diagnostic tests including PCR, ELISA or CAA tests can be used on NHPs [76], the sampling procedure whether blood or feces is complex. Third, administering praziquantel treatment to these animals is not trivial. An attempt to control chimpanzee infected populations by supplying praziquantel by food supply did not succeed and more complex administration procedure using gastric tube under anesthesia where preconized [75].

## Supporting information

S1 Table Natural host spectrum of *Schistosoma mansoni*. (DOCX)

S2 Table MS Database. (XLSX)

S3 Table Genetic diversity indices (He, Ar, Fis and number of breeders) for each infrapopulation. (DOCX)

S4 Table Results from general linear models examining the association of parasite population genetic indexes (He, Ar, Number of breeders and Fis) with host variables (sex and age). (DOCX)

S1 Fig Population structures of *Schistosoma mansoni* miracidia using Graphic 'deltaK'. (TIFF)

## References

[1] Herricks JR, Hotez PJ, Wanga V, Coffeng LE, Haagsma JA, Basanez MG, et al The global burden of disease study 2013: What does it mean for the NTDs? PLoS Neglected Tropical Diseases. 2017;11(8):e0005424 10.1371/journal.pntd.0005424 28771480 PMC5542388

[2] Rollinson D, Simpson AJG. The biology of Schistosomes. From genes to latrines. London: Academic Press; 1987. 446 p.

[3] Gryseels B, Polman K, Clerinx J, Kestens L. Human schistosomiasis. Lancet. 2006;368(9541):1106–18. Epub 2006/09/26. 10.1016/S0140-6736(06)69440-3 . 16997665

[4] Qian C, Zhang Y, Zhang X, Yuan C, Gao Z, Yuan H, et al Effectiveness of the new integrated strategy to control the transmission of *Schistosoma* japonicum in China: a systematic review and meta-analysis. Parasite. 2018;25:54 Epub 2018/11/18. 10.1051/parasite/2018058 30444486 PMC6238655

[5] Standley CJ, Dobson AP, Stothard JR. Out of Animals and Back Again: Schistosomiasis as a Zoonosis in Africa. 2012.

[6] Standley CJ, Mugisha L, Dobson AP, Stothard JR. Zoonotic schistosomiasis in non-human primates: past, present and future activities at the human-wildlife interface in Africa. Journal of Helminthology. 2012;86(2):131–40. 10.1017/S0022149X12000028 . 22269859

[7] Stothard JR, Mugisha L, Standley CJ. Stopping schistosomes from 'monkeying-around' in chimpanzees. Trends in Parasitology. 2012;28(8):320–6. Epub 2012/06/29. 10.1016/j.pt.2012.05.007 . 22738857

[8] Martins AV. Non-human vertebrate hosts of *Schistosoma haematobium* and *Schistosoma mansoni*. Bulletin of the World Health Organization. 1958;18(5–6):931–44. 13573118 PMC2537966

[9] Stirewalt MA, Kuntz RE, Evans AS. The relative susceptibilities of the commonly-used laboratory mammal to infection by *Schistosoma mansoni*. The American Journal of Tropical Medicine and Hygiene. 1951;31(1):57–82. Epub 1951/01/01. 10.4269/ajtmh.1951.s1-31.57 . 14799717

[10] Sadun EH, Von Lichtenberg F, Hickman RL, Bruce JI, Smith JH, Schoenbechler MJ. Schistosomiasis mansoni in the chimpanzee: parasitologic, clinical, serologic, pathologic and radiologic observations. The American Journal of Tropical Medicine and Hygiene. 1966;15(4):496–506. Epub 1966/07/01. 10.4269/ajtmh.1966.15.496 . 5941172

[11] Richards L, Erko B, Ponpetch K, Ryan SJ, Liang S. Assessing the nonhuman primate reservoir of *Schistosoma mansoni* in Africa: a systematic review. Infectious Disease of Poverty. 2019;8(1):32 Epub 2019/05/12. 10.1186/s40249-019-0543-7 31077256 PMC6509776

[12] Teklemariam D, Legesse M, Degarege A, Liang S, Erko B. *Schistosoma mansoni* and other intestinal parasitic infections in schoolchildren and vervet monkeys in Lake Ziway area, Ethiopia. BMC Research Notes. 2018;11(1):146 10.1186/s13104-018-3248-2 29463304 PMC5819654

[13] Fuller GK, Lemma A, Haile T. Schistosomiasis in man and monkeys in Omo National Park, southwest Ethiopia. Transactions of the Royal Society of Tropical Medicine and Hygiene. 1979;73(1):121–2. 10.1016/0035-9203(79)90149-4 . 108822

[14] Cheever AW, Kirschstein RL, Reardon LV. S*chistosoma mansoni* infection of presumed natural origin in Cercopithecus monkeys from Tanzania and Ethiopia. Bulletin of the World Health Organization. 1970;42(3):486–90. 5310215 PMC2427536

[15] Ouma J, Fenwick A. Animal reservoirs of schistosomiasis. In Macpherson CNL & Craig PS (Eds), Parasitic Helminths and Zoonoses in Africa 1991:224–36.

[16] Else JG, Satzger M. Natural infections of *Schistosoma mansoni* and *S*. *haematobium* in Cercopithecus monkeys in Kenya. Annals of Tropical Medicine and Parasitology. 1982;76(1):111–2. 10.1080/00034983.1982.11687512 . 7082073

[17] Muriuki SM, Murugu RK, Munene E, Karere GM, Chai DC. Some gastro-intestinal parasites of zoonotic (public health) importance commonly observed in old world non-human primates in Kenya. Acta Trop. 1998;71(1):73–82. 10.1016/s0001-706x(98)00040-0 . 9776144

[18] Pourrut X, Diffo JL, Somo RM, Bilong Bilong CF, Delaporte E, LeBreton M, et al Prevalence of gastrointestinal parasites in primate bushmeat and pets in Cameroon. Veterinary Parasitology. 2011;175(1–2):187–91. 10.1016/j.vetpar.2010.09.023 . 20970258

[19] Mbaya AW, Udendeye UJ. Gastrointestinal parasites of captive and free-roaming primates at the Afi Mountain Primate Conservation Area in Calabar, Nigeria and their zoonotic implications. Pakistan Journal of Biological Sciences: PJBS. 2011;14(13):709–14. 10.3923/pjbs.2011.709.714 . 22308652

[20] McGrew WC, TCE G., Collins DA, File SK. Intestinal parasites of sympatric Pan troglodytes and Papio Spp. at two sites: Gombe (Tanzania) and Mt. Assirik (Senegal) American Journal of Primatology. 1989;80(2):147–55. 10.1002/ajp.1350170204 31968849

[21] Ghandour AM, Zahid NZ, Banaja AA, Kamal KB, Bouq AI. Zoonotic intestinal parasites of hamadryas baboons Papio hamadryas in the western and northern regions of Saudi Arabia. The American Journal of Tropical Medicine and Hygiene. 1995;98(6):431–9. . 8544227

[22] Zahed NZ, Ghandour AM, Banaja AA, Banerjee RK, Dehlawi MS. Hamadryas baboons Papio hamadryas as maintenance hosts of *Schistosoma mansoni* in Saudi Arabia. Tropical Medicine and International Health. 1996;1(4):449–55. Epub 1996/08/01. 10.1046/j.1365-3156.1996.d01-100.x . 8765452

[23] Legesse M, Erko B. Zoonotic intestinal parasites in Papio anubis (baboon) and Cercopithecus aethiops (vervet) from four localities in Ethiopia. Acta Trop. 2004;90(3):231–6. 10.1016/j.actatropica.2003.12.003 . 15099809

[24] Murray S, Stem C, Boudreau B, Goodall J. Intestinal parasites of baboons (Papio cynocephalus anubis) and chimpanzees (Pan troglodytes) in Gombe National Park. Journal of Zoo and Wildlife Medicine. 2000;31(2):176–8. 10.1638/1042-7260(2000)031[0176:IPOBPC]2.0.CO;2 . 10982128

[25] Muller-Graf CD, Collins DA, Packer C, Woolhouse ME. *Schistosoma mansoni* infection in a natural population of olive baboons (Papio cynocephalus anubis) in Gombe Stream National Park, Tanzania. Parasitology. 1997;115 (Pt 6):621–7. 10.1017/s0031182097001698 . 9488873

[26] Fenwick A. Baboons as reservoir hosts of *Schistosoma mansoni*. Transactions of the Royal Society of Tropical Medicine and Hygiene. 1969;63(5):557–67. 10.1016/0035-9203(69)90172-2 . 5387893

[27] Munene E, Otsyula M, Mbaabu DA, Mutahi WT, Muriuki SM, Muchemi GM. Helminth and protozoan gastrointestinal tract parasites in captive and wild-trapped African non-human primates. Veterinary Parasitology. 1998;78(3):195–201. 10.1016/s0304-4017(98)00143-5 . 9760061

[28] Miller JH. Papio doguera (dog face baboon), a primate reservoir host of *Schistosoma mansoni* in East Africa. Transactions of the Royal Society of Tropical Medicine and Hygiene. 1960;54:44–6. 10.1016/0035-9203(60)90211-x . 14422536

[29] Hahn NE, Proulx D, Muruthi PM, Alberts S, Altmann J. Gastrointestinal parasites in free-ranging Kenyan baboons (*Papio cynocephalus* and *P*. *anubis*). International Journal of Primatology 2003;24(2):271–9.

[30] Weyher AH, Ross AG, Semple S. Gastrointestinal Parasites in Crop Raiding and Wild Foraging *Papio anubis* in Nigeria. International Journal of Primatology. 2006;27(6):1519–34.

[31] Lu DB, Wang TP, Rudge JW, Donnelly CA, Fang GR, Webster JP. Evolution in a multi-host parasite: chronobiological circadian rhythm and population genetics of *Schistosoma japonicum* cercariae indicates contrasting definitive host reservoirs by habitat. International Journal for Parasitology. 2009;39(14):1581–8. 10.1016/j.ijpara.2009.06.003 . 19577571

[32] Biek R, Real LA. The landscape genetics of infectious disease emergence and spread. Molecular Ecology. 2010;19(17):3515–31. Epub 2010/07/14. 10.1111/j.1365-294X.2010.04679.x 20618897 PMC3060346

[33] Wang TP, Shrivastava J, Johansen MV, Zhang SQ, Wang FF, Webster JP. Does multiple hosts mean multiple parasites? Population genetic structure of *Schistosoma japonicum* between definitive host species. International Journal for Parasitology. 2006;36(12):1317–25. 10.1016/j.ijpara.2006.06.011 . 16876170

[34] Rudge JW, Carabin H, Balolong E, Tallo V, Shrivastava J, Lu DB, et al Population genetics of *Schistosoma japonicum* within the Philippines suggest high levels of transmission between humans and dogs. PLoS Neglected Tropical Diseases. 2008;2(11):e340 10.1371/journal.pntd.0000340 . 19030225 PMC2582952

[35] Rudge JW, Lu DB, Fang GR, Wang TP, Basanez MG, Webster JP. Parasite genetic differentiation by habitat type and host species: molecular epidemiology of *Schistosoma japonicum* in hilly and marshland areas of Anhui Province, China. Molecular Ecology. 2009;18(10):2134–47. 10.1111/j.1365-294X.2009.04181.x . 19389178

[36] Chitsulo L, Engels D, Montresor A, Savioli L. The global status of schistosomiasis and its control. Acta Trop. 2000;77(1):41–51. 10.1016/s0001-706x(00)00122-4 . 10996119 PMC5633072

[37] Kebede T, Negash Y, Erko B. *Schistosoma mansoni* infection in human and nonhuman primates in selected areas of Oromia Regional State, Ethiopia. Journal of Vector Borne Diseases. 2018;55(2):116–21. Epub 2018/10/04. 10.4103/0972-9062.242558 . 30280709

[38] Erko B, Gebre-Michael T, Balcha F, Gundersen SG. Implication of Papio anubis in the transmission of intestinal schistosomiasis in three new foci in Kime area, Ethiopia. Parasitology International. 2001;50(4):259–66. Epub 2001/11/24. 10.1016/s1383-5769(01)00090-3 . 11719112

[39] Dufera M, Petros B, Erko B, Berhe N, Gundersen SG. *Schistosoma mansoni* Infection in Finchaa Sugar Estate: Public health Problem Assessment based on Clinical Records and Parasitological Surveys, Western Ethiopia. Sciences, Technology Arts Research Journal 2014;3:155–61.

[40] Gower CM, Shrivastava J, Lamberton PH, Rollinson D, Webster BL, Emery A, et al Development and application of an ethically and epidemiologically advantageous assay for the multi-locus microsatellite analysis of *Schistosoma mansoni*. Parasitology. 2007;134(Pt 4):523–36. 10.1017/S0031182006001685 . 17096873 PMC2613677

[41] Walsh PS, Metzger DA, Higuchi R. Chelex 100 as a medium for simple extraction of DNA for PCR-based typing from forensic material. BioTechniques. 1991;10(4):506–13. . 1867860

[42] Goudet J. FSTAT, a Program to Estimate and Test Gene Diversities and Fixation Indices (Version 2.9.3). http://wwwunilch/izea/softwares/fstathtml 2001.

[43] Curtis J, Sorensen RE, Page LK, Minchella DJ. Microsatellite loci in the human blood fluke *Schistosoma mansoni* and their utility for other schistosome species. Molecular Ecology Notes. 2001;1:143–5.

[44] Blair L, Webster JP, Barker GC. Isolation and characterization of polymorphic microsatellite markers in *Schistosoma mansoni* from Africa. Molecular Ecology Notes. 2001;1:93–5.

[45] Durand P, Sire C, Theron A. Isolation of microsatellite markers in the digenetic trematode *Schistosoma mansoni* from Guadeloupe island. Molecular Ecology. 2000;9(7):997–8. 10.1046/j.1365-294x.2000.00939-4.x . 10886664

[46] Blanton RE, Blank WA, Costa JM, Carmo TM, Reis EA, Silva LK, et al *Schistosoma mansoni* population structure and persistence after praziquantel treatment in two villages of Bahia, Brazil. International Journal for Parasitology. 41(10):1093–9. 10.1016/j.ijpara.2011.06.002 . 21784077 PMC3155667

[47] Silva LK, Liu S, Blanton RE. Microsatellite analysis of pooled *Schistosoma mansoni* DNA: an approach for studies of parasite populations. Parasitology. 2006;132(Pt 3):331–8. 10.1017/S0031182005009066 . 16255835

[48] Rodrigues NB, Coura Filho P, de Souza CP, Jannoti Passos LK, Dias-Neto E, Romanha AJ. Populational structure of *Schistosoma mansoni* assessed by DNA microsatellites. International Journal for Parasitol. 2002;32(7):843–51. 10.1016/s0020-7519(02)00031-0 . 12062555

[49] Jones OR, Wang J. COLONY: a program for parentage and sibship inference from multilocus genotype data. Molecular Ecology Resources. 2010;10(3):551–5. Epub 2011/05/14. 10.1111/j.1755-0998.2009.02787.x . 21565056

[50] Weir BS, Cockerham CC. Estimating F-Statistics for the Analysis of Population Structure. *Evolution*. 1984;38(6):1358–70 10.1111/j.1558-5646.1984.tb05657.x 28563791

[51] Pritchard JK, Stephens M, Donnelly P. Inference of population structure using multilocus genotype data. Genetics. 2000;155(2):945–59. 10835412 10.1093/genetics/155.2.945 PMC1461096

[52] Earl DA, vonHoldt BM. STRUCTURE HARVESTER: a website and program for visualizing STRUCTURE output and implementing the Evanno method. Conservation Genetics Resources. 2012;4(2):359–61.

[53] Evanno G, Regnaut S, Goudet J. Detecting the number of clusters of individuals using the software STRUCTURE: a simulation study. Molecular Ecology. 2005;14(8):2611–20. 10.1111/j.1365-294X.2005.02553.x . 15969739

[54] Aemero M, Boissier J, Climent D, Mone H, Mouahid G, Berhe N, et al Genetic diversity, multiplicity of infection and population structure of *Schistosoma mansoni* isolates from human hosts in Ethiopia. BMC Genetics. 2015;16:137 10.1186/s12863-015-0297-6 . 26630932 PMC4668696

[55] Agola LE, Mburu DN, DeJong RJ, Mungai BN, Muluvi GM, Njagi EN, et al Microsatellite typing reveals strong genetic structure of *Schistosoma mansoni* from localities in Kenya. Infection Genetics and Evolution. 2006;6(6):484–90. 10.1016/j.meegid.2006.03.002 . 16675308

[56] Thiele EA, Sorensen RE, Gazzinelli A, Minchella DJ. Genetic diversity and population structuring of *Schistosoma mansoni* in a Brazilian village. International Journal for Parasitology. 2008;38(3–4):389–99. 10.1016/j.ijpara.2007.07.011 . 17825305 PMC2476926

[57] Stothard JR, Webster BL, Weber T, Nyakaana S, Webster JP, Kazibwe F, et al Molecular epidemiology of *Schistosoma mansoni* in Uganda: DNA barcoding reveals substantial genetic diversity within Lake Albert and Lake Victoria populations. Parasitology. 2009;136(13):1813–24. 10.1017/S003118200999031X . 19627628

[58] Van den Broeck F, Maes GE, Larmuseau MH, Rollinson D, Sy I, Faye D, et al Reconstructing Colonization Dynamics of the Human Parasite *Schistosoma mansoni* following Anthropogenic Environmental Changes in Northwest Senegal. PLoS Neglected Tropical Diseases. 2015;9(8):e0003998 10.1371/journal.pntd.0003998 26275049 PMC4537236

[59] Gower CM, Gabrielli AF, Sacko M, Dembele R, Golan R, Emery AM, et al Population genetics of *Schistosoma haematobium*: development of novel microsatellite markers and their application to schistosomiasis control in Mali. Parasitology. 2011;138(8):978–94. 10.1017/S0031182011000722 . 21679489

[60] Gower CM, Gouvras AN, Lamberton PH, Deol A, Shrivastava J, Mutombo PN, et al Population genetic structure of *Schistosoma mansoni* and *Schistosoma haematobium* from across six sub-Saharan African countries: implications for epidemiology, evolution and control. Acta Trop. 2013;128(2):261–74. 10.1016/j.actatropica.2012.09.014 . 23041540

[61] Djuikwo-Teukeng F, Kouam Simo A, Alliene JF, Rey O, Njayou Ngapagna A, Tchuem Tchuente LA, et al Population genetic structure of *Schistosoma bovis* in Cameroon, Parasites & Vectors. 2019;12:56–67. 30678712 10.1186/s13071-019-3307-0 PMC6346511

[62] Curtis J, Sorensen RE, Minchella DJ. Schistosome genetic diversity: the implications of population structure as detected with microsatellite markers. Parasitology. 2002;125 Suppl:S51–9. 10.1017/s0031182002002020 . 12622328

[63] Curtis J, Minchella DJ. Schistosome population genetic structure: when clumping worms is not just splitting hairs. Parasitology today (Personal ed. 2000;16(2):68–71. Epub 2000/02/01. 10.1016/s0169-4758(99)01553-7 . 10652491

[64] Van den Broeck F, Meurs L, Raeymaekers JA, Boon N, Dieye TN, Volckaert FA, et al Inbreeding within human *Schistosoma mansoni*: do host-specific factors shape the genetic composition of parasite populations? Heredity (Edinb). 2014;113(1):32–41. 10.1038/hdy.2014.13 . 24619176 PMC4815646

[65] Meurs L, Mbow M, Boon N, van den Broeck F, Vereecken K, Dieye TN, et al Micro-geographical heterogeneity in *Schistosoma mansoni* and S. haematobium infection and morbidity in a co-endemic community in northern Senegal. PLoS Neglected Tropical Diseases. 2013;7(12):e2608 Epub 2014/01/05. 10.1371/journal.pntd.0002608 24386499 PMC3873272

[66] Meurs L, Mbow M, Vereecken K, Menten J, Mboup S, Polman K. Epidemiology of mixed *Schistosoma mansoni* and *Schistosoma haematobium* infections in northern Senegal. International Journal for Parasitology. 2012;42(3):305–11. 10.1016/j.ijpara.2012.02.002 . 22366733

[67] Caillaud D, Prugnolle F, Durand P, Theron A, de Meeus T. Host sex and parasite genetic diversity. Microbes & Infection. 2006;8(9–10):2477–83. Epub 2006/07/29. 10.1016/j.micinf.2006.06.003 . 16872857

[68] Boissier J, Chlichlia K, Digon Y, Ruppel A, Moné H. Preliminary study on sex-related inflammatory reactions in mice infected with *Schistosoma mansoni*. Parasitology Research. 2003;91(2):144–50. 10.1007/s00436-003-0943-1 12910415

[69] Nakazawa M, Fantappie MR, Freeman GL Jr., Eloi-Santos S, Olsen NJ, Kovacs WJ, et al *Schistosoma mansoni*: susceptibility differences between male and female mice can be mediated by testosterone during early infection. Expermntal Parasitology. 1997;85(3):233–40. 10.1006/expr.1997.4148 . 9085920

[70] Brouwer KC, Ndhlovu PD, Wagatsuma Y, Munatsi A, Shiff CJ. Urinary tract pathology attributed to *Schistosoma haematobium*: does parasite genetics play a role? The American Journal of Tropical Medicine and Hygiene. 2003;68(4):456–62. Epub 2003/07/24. . 12875296

[71] Smithers SR, Terry RJ. Resistance to experimental infection with *Schistosoma mansoni* in rhesus monkeys induced by the transfer of adult worms. Transactions of the Royal Society of Tropical Medicine and Hygiene. 1967;61(4):517–33. 10.1016/0035-9203(67)90102-2 4964088

[72] Smithers SR, Terry RJ. The immunology of schistosomiasis. Advances in parasitology. 1969;7:41–93. 10.1016/s0065-308x(08)60434-0 . 4998218

[73] Brown SP, Grenfell BT. An unlikely partnership: parasites, concomitant immunity and host defence. Proceedings of the Royal Society of London Series B Biological Sciences. 2001;268(1485):2543–9. 10.1098/rspb.2001.1821 . 11749708 PMC1088913

[74] Beltran S, Gourbal B, Boissier J, Duval D, Kieffer-Jaquinod S, Pierce RJ, et al Vertebrate host protective immunity drives genetic diversity and antigenic polymorphism in *Schistosoma mansoni*. Journal of Evolutionary Biology. 2011;24(3):554–72. 10.1111/j.1420-9101.2010.02190.x . 21159003

[75] Standley CJ, Mugisha L, Adriko M, Arinaitwe M, Rukundo J, Ajarova L, et al Intestinal schistosomiasis in chimpanzees on Ngamba Island, Uganda: observations on liver fibrosis, schistosome genetic diversity and praziquantel treatment. Parasitology. 2013;140(3):285–95. Epub 2012/10/26. 10.1017/S0031182012001576 . 23095137

[76] Standley CJ, Mugisha L, Verweij JJ, Adriko M, Arinaitwe M, Rowell C, et al Confirmed infection with intestinal schistosomiasis in semi-captive wild-born chimpanzees on Ngamba Island, Uganda. Vector Borne and Zoonotic Diseases (Larchmont, NY. 2011;11(2):169–76. Epub 2011/01/15. 10.1089/vbz.2010.0156 . 21231860

