# Supplementary material for: Genetic evidence for the role of non-human primates as reservoir hosts for human schistosomiasis
Source: PLoS Negl Trop Dis. 2020 Sep 8;14(9):e0008538. doi: 10.1371/journal.pntd.0008538 (PMC7500647; doi:10.1371/journal.pntd.0008538)
Supplement: S1 Table — (DOCX) [file pntd.0008538.s001.docx]

S1 table 1. Natural host spectrum of *Schistosoma mansoni*

| **Order** | **Family** | **species** | **Ref.** |
| --- | --- | --- | --- |
| Cetartiodactyla | Bovidae | *Bos spp* | **[1]** |
| Cetartiodactyla | Bovidae | *Kobus ellipsiprymnus* | **[2]** |
| Cetartiodactyla | Bovidae | *Ovis aries* | **[2]** |
| Rodentia | Caviidae | *Cavia aperea aperea* | **[1]** |
| Rodentia | Muridae | *Arvicanthis niloticus* | **[2]** |
| Rodentia | Muridae | Bolomys lasiurus | **[1]** |
| Rodentia | Muridae | *Dasymys incomtus* | **[2]** |
| Rodentia | Muridae | Gerbillus pyramidum | **[2]** |
| Rodentia | Muridae | *Holochilus brasiliensis* | **[1]** |
| Rodentia | Muridae | *Holochilus sciureus* | **[1]** |
| Rodentia | Muridae | *Lemniscomys griselda* | **[2]** |
| Rodentia | Muridae | *Lemniscomys rosalia* | **[1]** |
| Rodentia | Muridae | Lemniscomys striatus | **[3]** |
| Rodentia | Muridae | *Lophuromys flavopunctatus* | **[2]** |
| Rodentia | Muridae | *Mastomys huberti* | **[4]** |
| Rodentia | Muridae | Nectomys squamipes | **[1]** |
| Rodentia | Muridae | *Oenomys hypoxanthus* | **[2]** |
| Rodentia | Muridae | *Oryzomys nigripes* | **[5]** |
| Rodentia | Muridae | *Oryzomys squamipes* | **[6]** |
| Rodentia | Muridae | *Oryzomys subflavus* | **[1]** |
| Rodentia | Muridae | *Oryzomys utiaritensis* | **[1]** |
| Rodentia | Muridae | *Otomys angoniensis* | **[2]** |
| Rodentia | Muridae | Oxymycterus angularis | **[1]** |
| Rodentia | Muridae | *Pelomys fallax* | **[2]** |
| Rodentia | Muridae | *Praomys natalensis* | **[2]** |
| Rodentia | Muridae | *Rattus norvegicus* | **[1]** |
| Rodentia | Muridae | Rattus rattus alexandrinus | **[2]** |
| Rodentia | Muridae | *Rattus rattus frugivorus* | **[1]** |
| Rodentia | Muridae | *Tatera robusta robusta* | **[7]** |
| Rodentia | Muridae | *Zygodontomys brachyurus* | **[5]** |
| Rodentia | Muridae | *Zygodontomys brevicauda* | **[1]** |
| Rodentia | Muridae | *Zygodontomys lasiurus* | **[8]** |
| Rodentia | Muridae | *Zygodontomys pixuna* | **[9]** |
| Primates | Cebidae | *Saimiri spp* | **[1]** |
| Primates | Cercopithecidae | *Cercopithecus aethiops aethiops* | **[1]** |
| Primates | Cercopithecidae | *Cercopithecus aethiops sabaeus* | **[1]** |
| Primates | Cercopithecidae | *Cercopithecus mitis* | **[10]** |
| Primates | Cercopithecidae | *Cercopithecus pygerethrus* | **[2]** |
| Primates | Cercopithecidae | *Erythrocebus patas* | **[1]** |
| Primates | Cercopithecidae | *Papio anubis* | **[2]** |
| Primates | Cercopithecidae | *Papio papio* | **[2]** |
| Primates | Cercopithecidae | Papio ursinus | **[2]** |
| Primates | Hominidae | Homo sapiens | **[2]** |
| Primates | Hominidae | Pan satyrus | **[11]** |
| Primates | Hominidae | *Pan troglodytes* | **[2]** |
| Carnivora | Canidae | *Canis familiaris* | **[2]** |
| Carnivora | Procyonidae | *Procyon carnivorus nigripes* | **[1]** |
| Insectivora | Soricidae | *Crocidura luna* | **[2]** |
| Insectivora | Soricidae | *Crocidura olivieri* | **[2]** |
| Didelmorphia | Didelphidae | *Didelphis albiventris* | **[1]** |
| Edentata | Myrmecophagidae | *Myrmecophaga tridactyla* | **[1]** |

1. Rollinson, D. and V.R. Southgate, *The genus Schistosoma : a taxonomic appraisal*, in *The Biology of schistosomes*. 1987, Academic Press. p. 1-26.

2. Pitchford, R.J., *A check list of definitive hosts exhibiting evidence of the genus Schistosoma Weinland, 1858 acquired naturally in Africa and the Middle East.* Journal of Helminthology, 1977. **51**: p. 229-252.

3. McMahon, J.E. and S.S. Baalawy, *A search for animal reservoirs of Schistosoma Mansoni in the Mwanza area of Tanzania.* East Afr Med J, 1967. **44**(8): p. 325-6.

4. Lapierre, J., et al., *[Complement to the epidemiologic study of the focus of Schistosoma mansoni bilharziasis in Kara (northern Togo)].* Bull Soc Pathol Exot, 1992. **85**(3): p. 232-7.

5. Dias, L.C., F.D. Avila-Pires, and A.C. Pinto, *Parasitological and ecological aspects of Schistosomiasis mansoni in the valley of the Paraiba do Sul River (Sao Paulo State, Brazil) I. Natural infection of small mammals with Schistosoma mansoni.* Trans R Soc Trop Med Hyg, 1978. **72**(5): p. 496-500.

6. Silva, T.M. and Z.A. Andrade, *[Natural infection of wild rodents by Schistosoma mansoni].* Mem Inst Oswaldo Cruz, 1989. **84**(2): p. 227-35.

7. Mac Pherson, C.N.L. and P.S. Craig, *Parasitic helminths and zoonoses in Africa*. 1991, London: Unwin Hyman. 270.

8. Martins, V.A., G. Martins, and R.S. Siebra De Brito, *Reservatorios silvestres do Schistosoma mansoni no estado de Minas Gerais.* Revista Brasileira de Malariologia e Doencas Tropicais, 1955. **7**: p. 259-265.

9. Amorin, J.P., *Roedores selvagens como disseminadores de ovos de Schistosoma mansoni.* Revista do Instituto de Medicina Tropical de Sao Paulo, 1962. **4**: p. 397-402.

10. Nelson, G.S., C. Teesdale, and R.B. Highton, *The role of animals as reservoirs in Africa. In CIBA Foundation Symposium on Bilharziasis. G.E.W. Wolstenhome & M. O'Connor (eds), 149-227. London: Churchill.* 1962.

11. Hsu, S.Y. and H.F. Hsu, *A chimpanzee naturally infected with Schistosoma mansoni; its resistance against a challenge infection of S. japonicum.* Trans R Soc Trop Med Hyg, 1968. **62**(6): p. 901-2.
