# Supplementary material for: Genetic evidence for the role of non-human primates as reservoir hosts for human schistosomiasis
Source: PLoS Negl Trop Dis. 2020 Sep 8;14(9):e0008538. doi: 10.1371/journal.pntd.0008538 (PMC7500647; doi:10.1371/journal.pntd.0008538)
Supplement: S3 Table — (DOCX) [file pntd.0008538.s003.docx]

S2 Table 2

Genetic diversity indices (He, Ar, Fis and number of breeders) for each infrapopulation

| **Code** | **Sex** | **Age** | **He** | **Ar** | **Fis** | **Number of breeders** |
| --- | --- | --- | --- | --- | --- | --- |
| Bochesa human |  |  |  |  |  |  |
| BH1 | M | 20 | 0.53 | 1.53 | 0.078 | 47 |
| BH6 | M | 12 | 0.49 | 1.49 | 0.094 | 176 |
| BH7 | M | 28 | 0.52 | 1.52 | 0.094 | 163 |
| BH8 | M | 23 | 0.45 | 1.45 | 0.111 | 93 |
| BH9 | M | 16 | 0.49 | 1.49 | 0.049 | 81 |
| BH10 | M | 13 | 0.56 | 1.56 | 0.122 | 124 |
| BH11 | M | 15 | 0.52 | 1.52 | 0.050 | 76 |
| BH12 | M | 14 | 0.53 | 1.53 | 0.132 | 155 |
| BH13 | M | 18 | 0.52 | 1.52 | 0.137 | 233 |
| BH14 | M | 10 | 0.54 | 1.54 | 0.121 | 47 |
| BH15 | M | 36 | 0.56 | 1.56 | 0.116 | 248 |
| BH16 | M | 25 | 0.52 | 1.52 | 0.116 | 110 |
| BH17 | M | 10 | 0.53 | 1.53 | 0.016 | 108 |
| BH18 | M | 30 | 0.54 | 1.54 | 0.069 | NC |
| BH19 | M | 26 | 0.52 | 1.52 | 0.092 | 133 |
| BH20 | M | 18 | 0.54 | 1.54 | 0.128 | 127 |
| BH21 | M | 26 | 0.53 | 1.53 | 0.077 | 203 |
| BH22 | M | 16 | 0.55 | 1.55 | 0.117 | 88 |
| BH2 | F | 30 | 0.50 | 1.50 | 0.115 | 48 |
| BH3 | F | 7 | 0.55 | 1.55 | -0.002 | 133 |
| BH4 | F | 30 | 0.49 | 1.49 | 0.097 | 151 |
| BH5 | F | 13 | 0.51 | 1.51 | 0.014 | 37 |
| Bochesa Monkey | |  |  |  |  |  |
| BM67 |  |  | 0.52 | 1.52 | 0.100 | 26 |
| BM68 |  |  | 0.44 | 1.44 | -0.142 | 10 |
| BM69 |  |  | 0.48 | 1.48 | 0.013 | 79 |
| BM70 |  |  | 0.51 | 1.51 | -0.060 | 20 |
| BM71 |  |  | 0.46 | 1.46 | -0.022 | 32 |
| BM72 |  |  | 0.53 | 1.53 | -0.038 | 31 |
| BM73 |  |  | 0.54 | 1.55 | -0.087 | NC |
| BM74 |  |  | 0.55 | 1.55 | 0.006 | 37 |
| BM75 |  |  | 0.61 | 1.60 | 0.039 | 105 |
| BM76 |  |  | 0.52 | 1.52 | 0.107 | 37 |
| BM77 |  |  | 0.48 | 1.48 | -0.014 | 32 |
| BM78 |  |  | 0.50 | 1.50 | -0.066 | NC |
| BM79 |  |  | 0.46 | 1.39 | 0.077 | NC |
| Kime human |  |  |  |  |  |  |
| KH49 | M | 10 | 0.42 | 1.42 | 0.099 | 108 |
| KH50 | M | 15 | 0.40 | 1.40 | 0.179 | 43 |
| KH53 | M | 12 | 0.40 | 1.40 | 0.074 | 84 |
| KH58 | M | 9 | 0.38 | 1.38 | 0.163 | 75 |
| KH59 | M | 2 | 0.40 | 1.40 | 0.236 | 46 |
| KH62 | M | 11 | 0.42 | 1.42 | 0.007 | 48 |
| KH65 | M | 6 | 0.41 | 1.41 | 0.044 | 108 |
| KH51 | F | 12 | 0.41 | 1.41 | 0.080 | 120 |
| KH52 | F | 9 | 0.42 | 1.42 | 0.132 | 116 |
| KH54 | F | 5 | 0.41 | 1.41 | 0.022 | 101 |
| KH55 | F | 8 | 0.38 | 1.38 | 0.156 | 29 |
| KH56 | F | 25 | 0.41 | 1.41 | -0.081 | 34 |
| KH57 | F | 10 | 0.40 | 1.40 | 0.098 | 78 |
| KH60 | F | 12 | 0.39 | 1.39 | 0.027 | 50 |
| KH61 | F | 6 | 0.45 | 1.44 | 0.086 | 67 |
| KH63 | F | 28 | 0.40 | 1.40 | 0.090 | 33 |
| KH64 | F | 20 | 0.33 | 1.33 | 0.161 | NC |
| KH66 | F | 35 | 0.42 | 1.42 | 0.010 | 81 |
| Kime baboon |  |  |  |  |  |  |
| KB23 |  |  | 0.41 | 1.41 | 0.150 | 91 |
| KB24 |  |  | 0.44 | 1.44 | 0.177 | 136 |
| KB25 |  |  | 0.38 | 1.38 | 0.091 | 84 |
| KB26 |  |  | 0.41 | 1.41 | 0.136 | 55 |
| KB27 |  |  | 0.39 | 1.39 | 0.118 | 163 |
| KB28 |  |  | 0.41 | 1.41 | -0.036 | 66 |
| KB29 |  |  | 0.41 | 1.41 | 0.148 | 101 |
| KB30 |  |  | 0.41 | 1.41 | 0.203 | 79 |
| KB31 |  |  | 0.44 | 1.44 | 0.093 | 91 |
| KB32 |  |  | 0.40 | 1.40 | 0.130 | 109 |
| KB33 |  |  | 0.41 | 1.41 | 0.154 | 145 |
| KB34 |  |  | 0.42 | 1.42 | 0.145 | NC |
| KB35 |  |  | 0.40 | 1.40 | 0.142 | 62 |
| KB36 |  |  | 0.44 | 1.44 | 0.157 | 79 |
| KB37 |  |  | 0.42 | 1.42 | 0.140 | 70 |
| KB38 |  |  | 0.40 | 1.39 | 0.125 | 174 |
| KB39 |  |  | 0.40 | 1.40 | 0.051 | 102 |
| KB40 |  |  | 0.41 | 1.41 | 0.035 | 90 |
| KB41 |  |  | 0.42 | 1.42 | 0.193 | 109 |
| KB42 |  |  | 0.41 | 1.41 | 0.098 | 109 |
| KB43 |  |  | 0.41 | 1.41 | 0.076 | 140 |
| KB44 |  |  | 0.40 | 1.40 | 0.114 | 102 |
| KB45 |  |  | 0.40 | 1.40 | 0.032 | 105 |
| KB46 |  |  | 0.37 | 1.37 | 0.101 | 52 |
| KB47 |  |  | 0.43 | 1.43 | 0.108 | 240 |
| KB48 |  |  | 0.39 | 1.39 | 0.086 | 79 |
| Fincha human |  |  |  |  |  |  |
| FH81 | M | 21 | 0.57 | 1.57 | -0.014 | 92 |
| FH83 | M | 5 | 0.51 | 1.51 | -0.043 | NC |
| FH84 | M | 12 | 0.55 | 1.55 | 0.023 | 127 |
| FH85 | M | 9 | 0.54 | 1.54 | -0.031 | 184 |
| FH87 | M | 10 | 0.57 | 1.57 | 0.035 | 116 |
| FH88 | M | 10 | 0.54 | 1.54 | 0.091 | 127 |
| FH89 | M | 8 | 0.53 | 1.53 | 0.096 | 57 |
| FH90 | M | 7 | 0.55 | 1.55 | 0.047 | 124 |
| FH91 | M | 8 | 0.54 | 1.54 | -0.001 | 200 |
| FH93 | M | 11 | 0.54 | 1.54 | -0.061 | NC |
| FH94 | M | 25 | 0.55 | 1.56 | -0.146 | NC |
| FH80 | F | 8 | 0.57 | 1.57 | 0.075 | 61 |
| FH82 | F | 12 | 0.56 | 1.56 | 0.052 | 79 |
| FH86 | F | 7 | 0.57 | 1.57 | 0.023 | 95 |
| FH92 | F | 9 | 0.57 | 1.57 | 0.016 | 69 |
| FH95 | F | 10 | 0.52 | 1.52 | 0.012 | 145 |
| FH96 | F | 10 | 0.53 | 1.53 | -0.007 | 109 |
| FH97 | F | 8 | 0.58 | 1.57 | 0.095 | NC |
| FH98 | F | 9 | 0.47 | 1.46 | 0.051 | NC |
| FH99 | F | 32 | 0.77 | 1.50 | NA | NC |
| Fincha baboon |  |  |  |  |  |  |
| FB101 |  |  | 0.53 | 1.53 | 0.093 | 130 |
| FB102 |  |  | 0.53 | 1.53 | 0.047 | 117 |
| FB103 |  |  | 0.56 | 1.56 | 0.039 | 66 |
| FB104 |  |  | 0.48 | 1.47 | 0.086 | NC |
| FB105 |  |  | 0.59 | 1.59 | 0.035 | NC |
|  |  |  |  |  |  |  |
| NC: Not calculated because the number of miracidia <10 | | | | |  |  |
