# Supplementary material for: Genetic evidence for the role of non-human primates as reservoir hosts for human schistosomiasis
Source: PLoS Negl Trop Dis. 2020 Sep 8;14(9):e0008538. doi: 10.1371/journal.pntd.0008538 (PMC7500647; doi:10.1371/journal.pntd.0008538)
Supplement: S4 Table — (DOCX) [file pntd.0008538.s004.docx]

S3 Table 3

Results from general linear models examining the association of parasite population genetic indexes (He, Ar, Number of breeders and Fis) with host variables (sex and age)

|  |  | **Slope** | **Standard Error** | **P-value** |
| --- | --- | --- | --- | --- |
| **He** | *Intercept* | 0.4782699 | 0.0285020 | <2e-16 *** |
|  | *Age* | 0.0003705 | 0.0016284 | 0.821 |
|  | *Sex* | -0.0029754 | 0.0394177 | 0.940 |
|  | *Age * Sex* | 0.0017308 | 0.0022786 | 0.451 |
| **Ar** | *Intercept* | 1.496962 | 0.023956 | <2e-16 *** |
|  | *Age* | -0.001738 | 0.001369 | 0.2093 |
|  | *Sex* | -0.022069 | 0.033130 | 0.5081 |
|  | *Age * Sex* | 0.003884 | 0.001915 | **0.0473 *** |
| **Number of Breeders** | *Intercept* | 91.4121 | 18.5038 | 9.88e-06 *** |
|  | *Age* | -0.6722 | 1.0881 | 0.53965 |
|  | *Sex* | -29.9682 | 25.6549 | 0.24852 |
|  | *Age * Sex* | 4.4217 | 1.5257 | **0.00564 **** |
| **Fis** | *Intercept* | 0.0566807 | 0.0272607 | 0.0423 * |
|  | *Age* | 0.0001125 | 0.0016434 | 0.9457 |
|  | *Sex* | 0.0095380 | 0.0372460 | 0.7988 |
|  | *Age * Sex* | 0.0001729 | 0.0022154 | 0.9381 |
