# Supplementary figures and images for: Genetic evidence for the role of non-human primates as reservoir hosts for human schistosomiasis
Source: PLoS Negl Trop Dis. 2020 Sep 8;14(9):e0008538. doi: 10.1371/journal.pntd.0008538 (PMC7500647; doi:10.1371/journal.pntd.0008538)

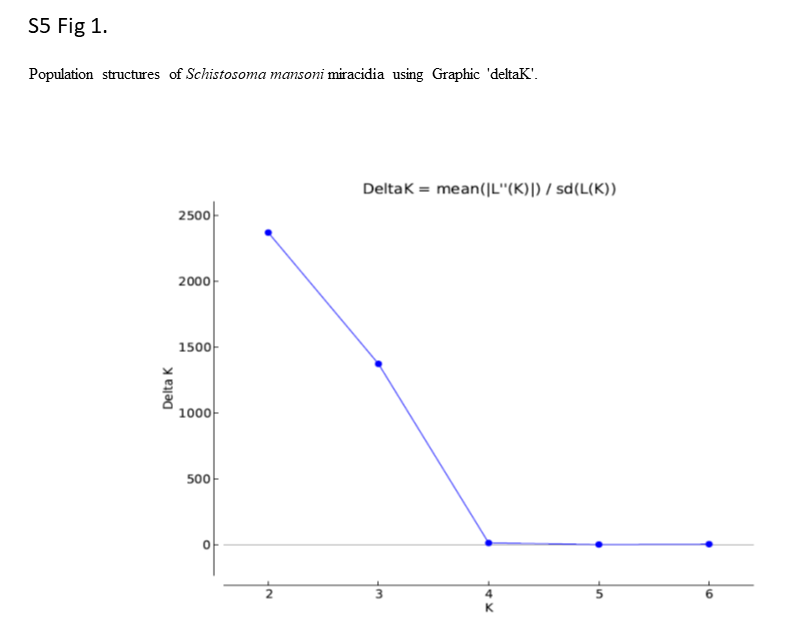

Supplement: S1 Fig — (TIFF) [file pntd.0008538.s005.tiff]
